# Editorial: Motor Correlates of Motivated Social Interactions

**DOI:** 10.3389/fpsyg.2022.858891

**Published:** 2022-03-18

**Authors:** John F. Stins, Miguel A. Muñoz, Thierry Lelard, Harold Mouras

**Affiliations:** ^1^Department of Human Movement Sciences, Faculty of Behavioural and Movement Sciences, Vrije Universiteit Amsterdam, Amsterdam Movement Sciences, Amsterdam, Netherlands; ^2^Centro de Investigación Mente, Cerebro y Comportamiento (CIMCYC), University of Granada, Granada, Spain; ^3^UR-UPJV 3300, Adaptations Physiologiques à l'Exercice et Réadaptation à l'Effort (EA 3300), UFR des Sciences du Sport, Université de Picardie Jules Verne, Amiens, France; ^4^UR-UPJV 4559, Laboratoire de Neurosciences Fonctionnelles et Pathologies, UFR de Médecine, Université de Picardie Jules Verne, Amiens, France

**Keywords:** social interactions, motor correlates, motivation, synchrony, affective neuroscience, social neuroscience

Facial expressions and bodily movements form an integral part of social interactions. When two (or more) individuals form a temporary alliance, such as engaging in a conversation, their facial and bodily movements often display synchronized behavior, implying some sort of spatial and temporal alignment. It is now recognized that synchronization may be the cornerstone of successful social interactions, perhaps even more so than messages conveyed in overt speech acts.

In the past, motor behaviors were often considered “peripheral” by psychologists, and hence hardly worthy of scientific investigation. For decades, the research emphasis has been on mental representations and the inner workings of the mind. With the emergence of more “embodied” perspectives on cognition, the bodily grounding of social, and emotional behavior has become more apparent.

As the interaction between motor and affective processes is fundamental to consider within the framework of motivated social interactions, it is important to understand both the interaction of these processes at the level of the individual but also at the dyadic level of the interaction, e.g., by explaining how different individuals interact and/or synchronize together in various contexts.

This Research Topic has brought together four contributions.

1. The study by Manders et al. investigated interactional synchrony in adult individuals on the autism spectrum. The authors analyzed video data obtained across sessions of dance/ movement therapy. Results of this (pilot) study involved rhythmic synchrony between actors, and affective engagement with a partner. The findings may have implications for creative arts therapies and movement-based interventions, emphasizing interactional synchrony, in individuals on the autism spectrum.
2. The study by Nyman-Salonen et al. investigated the synchrony of head and body movements in the framework of couple therapy with four participants (two spouses and two therapists). The authors analyzed couple therapy sessions using motion energy analysis (MEA), as well as self-reported wellbeing and therapeutic alliance. Results of this study showed significant synchrony between the majority of dyads. The findings show the importance of non-verbal synchrony (especially in-phase) in couple therapy as well as in individual psychotherapy.
3. Successful social interactions are often based on our perception of the faces of others, reflecting their behavioral intentions. Such information is determined not only by the facial expression itself, but also the direction of gaze (i.e., direct or deviated). Lebert et al. investigated the action tendencies in response to gaze direction of emotional faces. Action tendencies were investigated in terms of spontaneous postural adjustments, especially postural stability (sway area) and forward/backward displacements of the COP, perhaps reflective of approach-avoidance tendencies. Analysis of the COP yielded little effect of gaze and direction. However, postural parameters were correlated with personality traits, this could explain high variability reported in COP displacement between individuals.
4. The paper of Ayache et al. was a systematic review and theoretical treatment of interpersonal coordination. First, the authors established that “interpersonal coordination” has been used in various ways in the literature, making it an ill-defined concept. Next, they identified 19 studies on spontaneous motor interactions between two unacquainted healthy individuals. The review revealed a wide array of experimental paradigms, and tasks, e.g., conversations or collaborative problem solving. The paper demonstrates that the research field is rapidly expanding, but also plagued by conceptual and terminological difficulties.

The four papers of this Research Topic each provide a unique perspective on spontaneous motor interactions between two actors. All papers (and the citations therein) share the assumption that interpersonal coordination can act as “social glue” (a phrase used by Ayache et al.). This can have important implications for settings where social interaction is compromised, as in autism (Manders et al.) and therapy/counseling (Nyman-Salonen et al.). Another theme that emerges from these contributions is that movement patterns in a social context are often unintentional/unconscious, as for example spontaneous postural adjustments in response to facial expressions. No doubt, such spontaneous motor responses are likely registered by other actors, and this may result in episodes of unintentional interpersonal coordination, fostering further social interactions at different levels, such as verbal communication, the willingness to cooperate, shared problem solving, etc. In this sense, recent research showing the influence of idle movements (non-voluntary, non-systematically perceived movements specific to living beings, such as the blink of the eyes) on the quality of the interlocutor's perception and on the observer's postural adjustments (Treal et al., 2020, 2021) supports this hypothesis. Finally, the field is still in its infancy. We concur with Ayache et al. who state that at the beginning of their papers that “Studies of social interactions are situated at the crossroads of anthropology, sociology, philosophy, and psychology” (p. 2). We would like to add “physics” to this list, as many experimental paradigms are influenced by theories on spontaneous pattern formation and the emergence (and disappearance) of stable states in ensembles consisting of many interaction parts, such as muscles, neurons, limbs, etc. “Cognitive Neuroscience” should also be added thinking that the study of the motor correlates of social interactions concerns above all the study of neural mechanisms which are produced by the brain with the aim of establishing a social bond that is optimally adapted to the circumstances.

## Author Contributions

All authors listed have made a substantial, direct, and intellectual contribution to the work and approved it for publication.

## Conflict of Interest

The authors declare that the research was conducted in the absence of any commercial or financial relationships that could be construed as a potential conflict of interest.

## Publisher's Note

All claims expressed in this article are solely those of the authors and do not necessarily represent those of their affiliated organizations, or those of the publisher, the editors and the reviewers. Any product that may be evaluated in this article, or claim that may be made by its manufacturer, is not guaranteed or endorsed by the publisher.
